# TGF-β1 facilitates cell–cell communication in osteocytes via connexin43- and pannexin1-dependent gap junctions

**DOI:** 10.1038/s41420-019-0221-3

**Published:** 2019-10-25

**Authors:** Wenjing Liu, Demao Zhang, Xin Li, Liwei Zheng, Chen Cui, Yujia Cui, Jianxun Sun, Jing Xie, Xuedong Zhou

**Affiliations:** State Key Laboratory of Oral Diseases, West China Hospital of Stomatology, Sichuan University, Chengdu, China

**Keywords:** Cell growth, Biophysical chemistry

## Abstract

Connexins and pannexins are two families of channel forming proteins that are able to pass small molecules to achieve communication between cells. While connexins have been recognized to mediate gap junctional intercellular communication (GJIC), pannexins are far less known. Our previous study reported the potential role of TGF-β1 in mediating of connexins in osteocytes in vitro. Herein, we aimed to elucidate the influence of TGF-β1 on cell–cell communication based on gap junctions assembled by connexins and pannexins in vitro and ex vivo. We first showed that TGF-β1 positively affected the elongation of dendritic processes of osteocytes. Our data indicated that TGF-β1 increased expressions of connexin43 (Cx43) and pannexin1 (panx1), which are indispensable for hemichannel formation in gap junctions, in osteocytes in vitro and ex vivo. TGF-β1 enhanced gap junction formation and impacted cell–cell communication in living osteocytes, as indicated by the scrape loading and Lucifer yellow transfer assays. TGF-β1 enhanced the expressions of Cx43 and panx1 via activation of ERK1/2 and Smad3/4 signalling. The TGF-β1-restored expressions of Cx43 and panx1 in osteocytes in the presence of an ERK inhibitor, U0126, further demonstrated the direct participation of Smad3/4 signalling. TGF-β1 increased the accumulation of Smad3 in the nuclear region (immunofluorescence assay) and promoted the enrichment of Smad3 at the binding sites of the promoters of *Gja1* (Cx43) and *Panx1* (ChIP assay), thereby initiating the enhanced gene expression. These results provide a deep understanding of the molecular mechanisms involved in the modulation of cell–cell communication in osteocytes induced by TGF-β1.

## Introduction

Homeostasis depends on close connections and intimate molecular exchanges among extracellular, intracellular and intercellular networks. Direct communication between cells through specialized intercellular channels is completed by transmembrane channels in the plasma membranes between adjacent cells^1^. These channels are called gap junctions (GJ) and comprise connexin (Cx) proteins. The role of these channels is to allow the transfer of molecules smaller than 1.2 kDa between cells^2^. This flux is called gap junctional intercellular communication (GJIC) and is recognized as the basic mechanism in the transfer of accurate signals in response to external and internal stimuli^3^. To date, at least 20 and 21 different connexin genes have been respectively identified in mice and humans^4^. Among these Cx transmembrane proteins, connexin43 (Cx43) is the most abundant in bone^5^. Structurally, the 21 members of the connexin family have similar topological structures, including cytoplasmic N-, and C-terminal domains, along with four membrane spanning domains, two extracellular loops, and one intracellular loop^4,6^. Six connexin ligands are organized in a hexamer arrangement and transported to the cell surface to form a connexon, which is then paired with a partner connexon from a neighboring cell to form a gap junction channel^7^.

Pannexins (Panx), discovered in 2000, are a new set of channel proteins^5^. There are 3 pannexin genes, namely, Panx1, Panx2, and Panx3, which encode proteins strikingly similar to connexins in terms of structural topology. In recent years, Panx proteins have often been studied with connexins. Despite their structural similarities, pannexins have a unique character in that they function as unpaired channels^8^. Both Panx1 and Panx3 are located on the plasma membrane surface and in the endoplasmic reticulum. Panx2 has been found in the membranes of endosomal vesicles^9^. Panx1 and Panx3 are broadly expressed in skeletal cells^9–11^, whereas Panx2 seems to only be expressed in the nervous system^12^. Panx1 has been detected in murine osteoblasts^11^, whereas Panx1 has rarely been studied in osteoblasts or osteocytes^8^.

TGF-β1 is a multifunctional growth factor involved in various biological processes including cell proliferation, cell differentiation, tissue repair, inflammation, apoptosis, and cell motility^13^. TGF-β1 is well known to regulate cells of the skeletal muscle system in embryonic and adult organisms^14^. TGF-β1 has been demonstrated to modulate Cx43 expression and gap junction formations; this regulatory effect varies with different cell types^15^. Recently, studies have shown that TGF-β1 upregulates the expression of Cx43 in human granulosa cells^16^ and trophoblast cells^17^. In contrast, some studies have also reported that TGF-β1 downregulates connexin 43 expression and gap junction intercellular communication in rat hepatic stellate cells^18^ and inhibits Cx43 expression in smooth muscle cells from human detrusor^19^. TGF-β1/Smad3 signaling is assumed to be involved in the down-regulation of Cx43 in rats with sacral spinal cord injury^20^.

These paradoxical roles of TGF-β1 in regulating Cx43 expression in different cell types have prompted us to study the effects of TGF-β1 on Cx43 expression and GJIC in osteocytes. Moreover, to the best of our knowledge, there have been no reports on the effects of TGF-β1 on panx1 expression of osteocytes yet. We stimulated cultured osteocytes and those in bones with recombinant TGF-β1 and subsequently analyzed Cx43 and panx1 expression and the formation of functional GJs. We further explored the possible mechanism by which TGF-β1 modulates Cx43 and panx1.

## Results

### TGF-β1 increases the number of dendritic processes of osteocytes in bone tissue and MLO-Y4 cell line

**Fig. 1:**
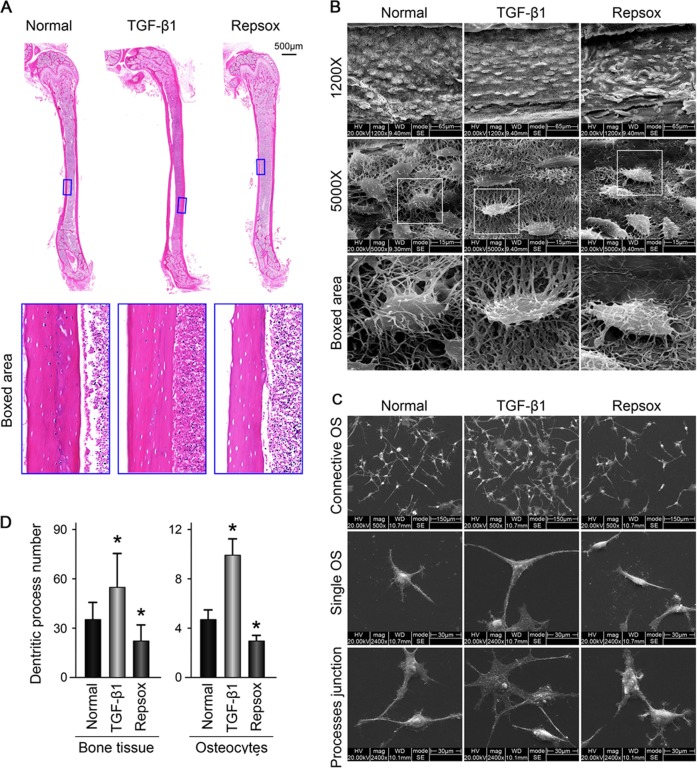
TGF-β1 promotes the number of dendritic processes of osteocytes in bone tissue and MLO-Y4 cell line. **a** Representative histological examination of cortical femora stained with HE showing lacuna size change in bone with TGF-β1 (10ng/ml) or its inhibitor Repsox (50 μM) after 21 days ex vivo culture (*n* = 6). **b** Representative SEM images revealing osteocyte canalicular system change in the cortex of mice treated with TGF-β1 (10 ng/ml) or Repsox (50 μM). Intact femurs were harvested from 4-week old mice and ex vivo cultured in 10% FBS DMEM with TGF-β1 or Repsox for 21 days (*n* = 6). Original magnification, ×1200 and ×5000. Details of the dendritic processes changes were shown in the boxed area (white). **c** Representative SEM images demonstrating the changes of dendritic processes of osteocytes (MLO-Y4 cell line) treated with TGF-β1 (5 ng/ml) or Repsox (25 μM). The cells were incubated with TGF-β1 or Repsox for 24 h. Accordingly, process junctions between cytoplasmic arms of osteocytes altered (lower lane). The results shown are based on three independent experiments (*n* = 3). **d** Quantitative analyses of the dendritic processes changes of osteocytes in **b**, **c**. Quantitative analyses were based on three independent experiments (*n* = 3). The results are shown as the mean ± s.d.; *n* = 3; *p < 0.05 by *t*-test

To explore the effects of TGF-β1 on communications between osteocytes, we first detected the osteocyte morphological changes induced by recombined TGF-β1 in cortical bones from mouse femurs ex vivo and in the MLO-Y4 cell line in vitro. Hematoxylin and eosin staining results showed no morphological changes in the cortical bones when induced by TGF-β1 (10 ng/ml) treatment for 21 days. However, the sizes of bone lacunae were observed to increase when induced by Repsox (50 μM), an inhibitor of TGF-β1 receptors (Fig. 1a).

We next used SEM to detect the three-dimensional organization of the osteocyte lacuna-canalicular system in cortical bone induced by TGF-β1 (Fig. 1b). The osteocytes formed interconnections with each other by dendritic processes. We found that the number of dendritic processes of osteocytes in cortical bone increased after TGF-β1 (10 ng/ml) treatment for 21 days. Conversely, in the presence of Repsox (50 μM), the number of dendritic processes decreased (see single cell in boxed area indicating variations in dendritic processes). To further confirm the changes in dendritic processes of individual cells and junctions between processes, we used osteocyte cell line MLO-Y4 in vitro (Fig. 1c). By SEM, it was found that TGF-β1 enhanced cell numbers and increased the dendritic processes of osteocytes; furthermore, the extracellular matrix (ECM) of the cells increased. The number of osteocytes decreased in the group treated with Repsox to levels lower than controls. Moreover, in the TGF-β1 group, more processes junctions were formed between neighboring osteocytes, whereas in the Repsox group, the processes junction formations weakened (lower lane in Fig. 1c). Quantification analyses on the numbers of dendritic processes confirmed these results (Fig. 1d).

### TGF-β1 promotes the expression of two channel-forming proteins of osteocyte, Cx43 and pannexin1

**Fig. 2:**
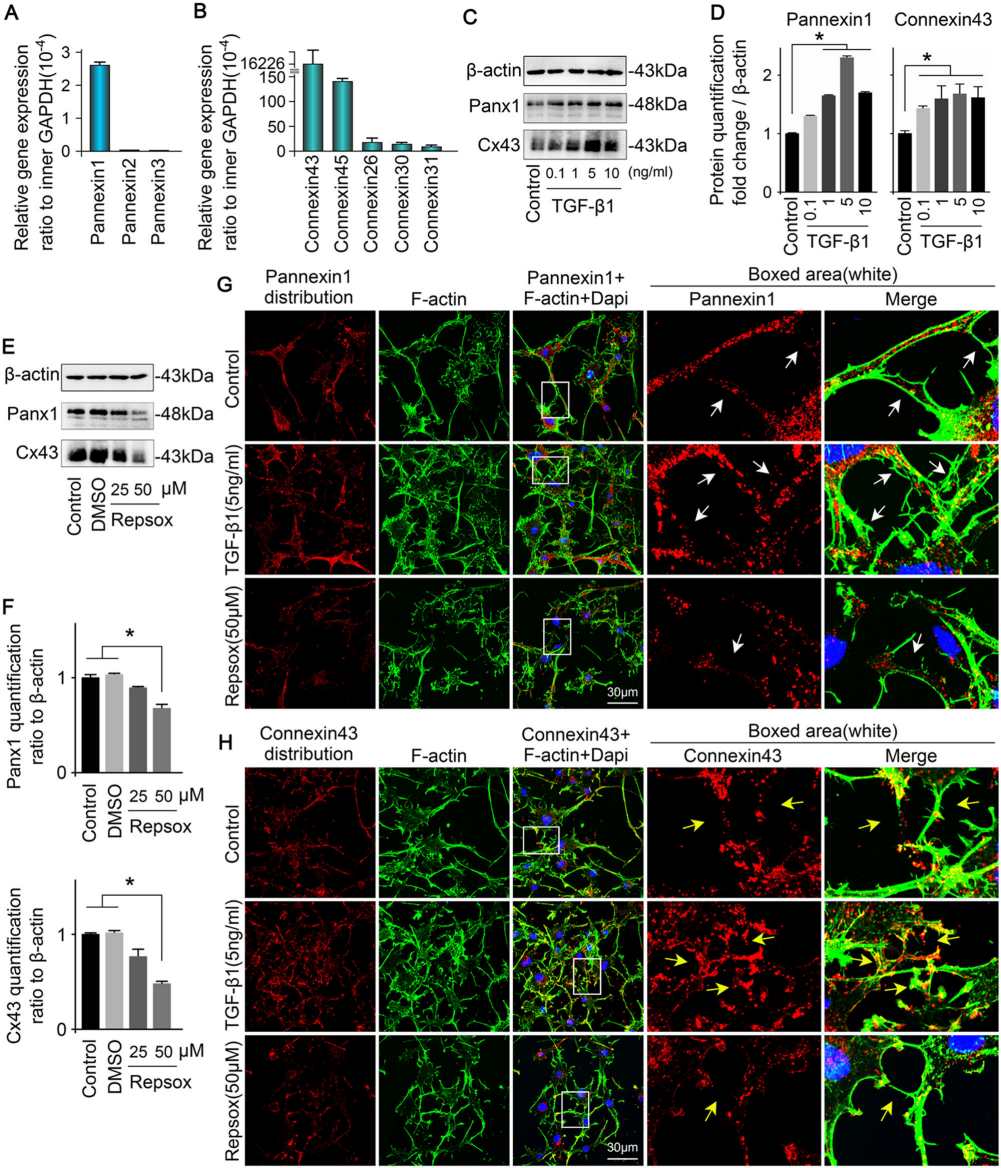
TGF-β1 enhances expression of Cx43 and pannexin1 of osteocytes. **a** Gene profile of pannexins in osteocytes (MLO-Y4 cell line). Quantitative real-time PCR showed that pannexin1 was detected in osteocytes, whereas pannexin2 and pannexin3 were hardly detected. The results shown are based on three independent experiments (*n* = 3). **b** Gene profile of connexins in osteocytes (MLO-Y4 cell line). Quantitative real-time PCR confirmed that gene expression of Cx43 were much higher than other connexins, such as Cx44, Cx26, Cx30, and Cx31. The results shown are based on three independent experiments (*n* = 3). **c** Western blots of protein lysates from osteocytes (MLO-Y4 cell line) reveals the enhanced expression of Cx43 and pannexin1 in osteocytes after treatment with TGF-β1 (0.1, 1, 5, 10 ng/ml). **d** Quantitative analysis was performed to confirm the protein changes in **c**. The results shown are based on three independent experiments (*n* = 3). Data are presented as the mean ± s.d.; *p < 0.05 by *t-*test. **e** Western blots of protein lysates from osteocytes (MLO-Y4 cell line) reveal reduced expressions of Cx43 and pannexin1 in osteocytes after treatment with Repsox (25, 50 μM). **f** Quantitative analysis was performed to confirm the protein changes in **e**. The results shown are based on three independent experiments (*n* = 3). Data are presented as the mean ± s.d.; *p < 0.05 by *t*-test. **g** Representative IF staining by CLSM showing the increased expression of pannexin1 in osteocytes in response to TGF-β1 (5 ng/ml) (cytoskeleton, green; panx1, red; nucleus, blue). The results shown are based on three independent experiments (*n* = 3). **h** Representative IF staining by CLSM showing the increased expression of Cx43 in osteocytes in response to TGF-β1 (5 ng/ml) (cytoskeleton, green; Cx43, red; nucleus, blue). The results shown are based on three independent experiments (*n* = 3)

We investigated the expressions of pannexin and connexin families (Table 1), which regulate gap junction formation and cell communication, in the MLO-Y4 cell line using quantitative real-time RCR (Fig. 2a, b). The results showed that panx1 exhibited the highest expression among pannexins whereas the other two pannexins had almost no expression (Fig. 2a). For connexins, we found that connexin43 was the most abundant in osteocytes (up to 16226 × 10^–4^-fold change compared with internal GAPDH control), whereas connexin44 (Cx44) expression was negligible (down to 143 × 10^–4^-fold compared with internal GAPDH control) (Fig. 2b). To examine the effects of TGF-β1 on Cx43 and panx1 protein expression in osteocytes, we cultured osteocytes with different concentration of TGF-β1 (0.1, 1, 5 and 10 ng/ml) and found that 5 ng/ml TGF-β1 significantly upregulated Cx43 and panx1 at 48 h by Western blot (Fig. 2c). In osteocytes induced by 5 ng/ml TGF-β1, the expression of panx1 was approximately 2.4-fold higher, whereas Cx43 was nearly 1.5-fold higher (Fig. 2d). To confirm the effects of TGF-β1 on Cx43 and panx1, we further administered Repsox (25 and 50 μM), an effective inhibitor of TGF-β type I receptor, on the MLO-Y4 for 48 h. The induced Cx43 and panx1 showed a dose-dependent decrease at 48 h (DMSO was used as the vehicle control), especially in the 50 μM Repsox group (Fig. 2e). Quantification further confirmed Repsox treatment decreased panx1 and Cx43 (Fig. 2f). To explore the distribution of Cx43 and pannexin1 in osteocytes induced by TGF-β1, we performed immunofluorescence staining (Fig. 2g, h). Dot-like distributions of panx1 and Cx43 were present in cell processes and cytoplasm, but were barely distributed in nuclei. After TGF-β1 (5 ng/ml) treatment, the expressions of panx1 and Cx43 were greatly enhanced and accompanied by increased numbers of dendritic processes. The high expressed panx1 and Cx43 clustered and even formed red fluorescent plaques along the cell processes. After the administration of Repsox (50 μM), reduced numbers of processes and lower fluorescent signals indicative of panx1 and Cx43 were observed. Furthermore, no clustering of these proteins was observed at the tips of the dendritic processes (details could be seen in the boxed area).

### TGF-β1 increases osteocyte communication through the activity of gap junctions formed by hemichannels assembled by connexin43 and pannexin1

**Fig. 3:**
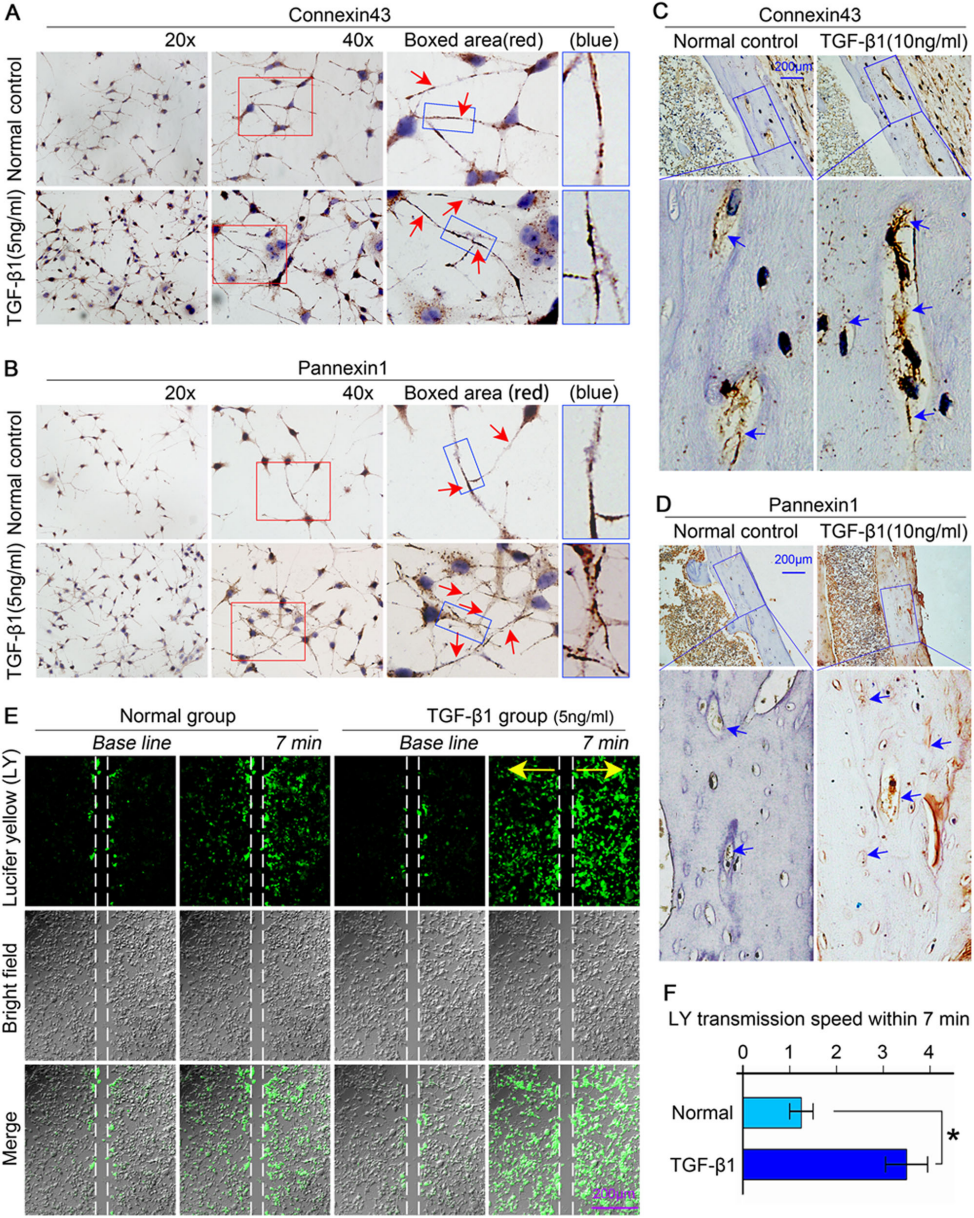
TGF-β1 promotes the connectivity between osteocytes via increasing the expression of Cx43 and pannexin1. **a**, **b** Representative images of immunohistochemistry (IHC) using the anti-Cx43 antibody (top) and anti-pannexin1 antibody (bottom) coupled with Gill’s haematoxylin counterstaining on cells cultured on glass slides showing increased expression of Cx43 and pannexin1 after TGF-β1 (5 ng/ml) treatment. Boxed area (red) shows that Cx43 and panx1 clustered to form plaques at the sites which eventually lead to gap junction formation. Additionally, panx1 also showed its expression on the membrane and in the cytoplasm at the tips of dendritic processes. The results shown are based on three independent experiments (*n* = 3). **c**, **d** Representative immunohistochemistry staining (IHC) images confirming the enhanced expression of Cx43 (up) and panx1 (down) in osteocytes residing in cortical bone of femur after treatment with TGF-β1(10 ng/ml). The results shown are based on three independent experiments (*n* = 3). **e** The Lucifer yellow (LY) coupling assay showing the increased transmission of dye (small fluorescent molecules) between living osteocytes (MLO-Y4 cell line) induced by TGF-β1 (5 ng/ml) by CLSM at ×40 magnification. The dye transmission images were obtained at 7 min after Lucifer yellow stain in the living osteocytes. The results shown are based on three independent experiments (*n* = 3). **f** Statistical analysis showing the different transmission speeds of Lucifer yellow between the control and TGF-β1 (5 ng/ml) group. Data are presented as the mean ± s.d.; n = 3, *p < 0.05 by *t-*test

IHC analysis further revealed increased levels of Cx43 and panx1 in osteocytes in vitro (Fig. 3a, b) and ex vivo (Fig. 3c, d) when induced by TGF-β1. In osteocytes in vitro, Cx43 primarily localized around nuclei and the dendritic processes. At the ends of the processes or at the junctions between the processes of adjacent cells, Cx43 expression was clustered as plaques (Fig. 3a, boxed area). Panx1-positive spots mainly distributed in the cytoplasm and at the edges of dendritic processes (Fig. 3b, boxed area). After TGF-β1 treatment (5 ng/ml) for 48 h, more Cx43-positive and panx1-positive spots were detected compared with controls (Fig. 3a, b). In osteocytes ex vivo, the Cx43-positive and panx1-positive areas were mainly distributed around lacunae edges as rings and in the matrix of cortical bone. After TGF-β1 treatment (10 ng/ml) for 21 days, the distributions of Cx43-positive (Fig. 3c) and panx1-positive (Fig. 3d) areas in osteocytes were significantly enhanced relative to those in normal controls (Fig. 3c, d, boxed areas). Additionally, TGF-β1 enhanced the accumulation of Cx43 and panx1 at the sites between adjacent osteocytes in the lacunae (Fig. 3c, d, boxed areas).

The accumulation of Cx43 and panx1 induced by TGF-β1 at the junction between the processes of adjacent osteocytes indicated increased hemichannel formation, which are indispensable for gap junctions. To explore cell–cell communications via gap junction activities between two osteocytes, scrape loading and dye transfer assays were performed in living osteocytes (Fig. 3e). After treatment with TGF-β1 (5 ng/ml) for 6 h, the transmission speed between the coupled living osteocytes was faster than that in a non-treated normal group 7 min after scrape loading. Quantification confirmed that the transmission speed induced by TGF-β1 was up to 2.8-fold greater compared with normal controls (Fig. 3f). The scrape loading and dye transfer assays indicated that more gap junctions formed at the terminals of dendritic processes between the adjacent coupling osteocytes when induced by TGF-β1; these increased gap junction numbers enabled the rapid transmission of Lucifer yellow molecules and thus promoted cell–cell communications.

### TGF-β1 triggers ERK1/2 and Smad3/4 signaling to mediate the expression of connexin43 and pannexin1

**Fig. 4:**
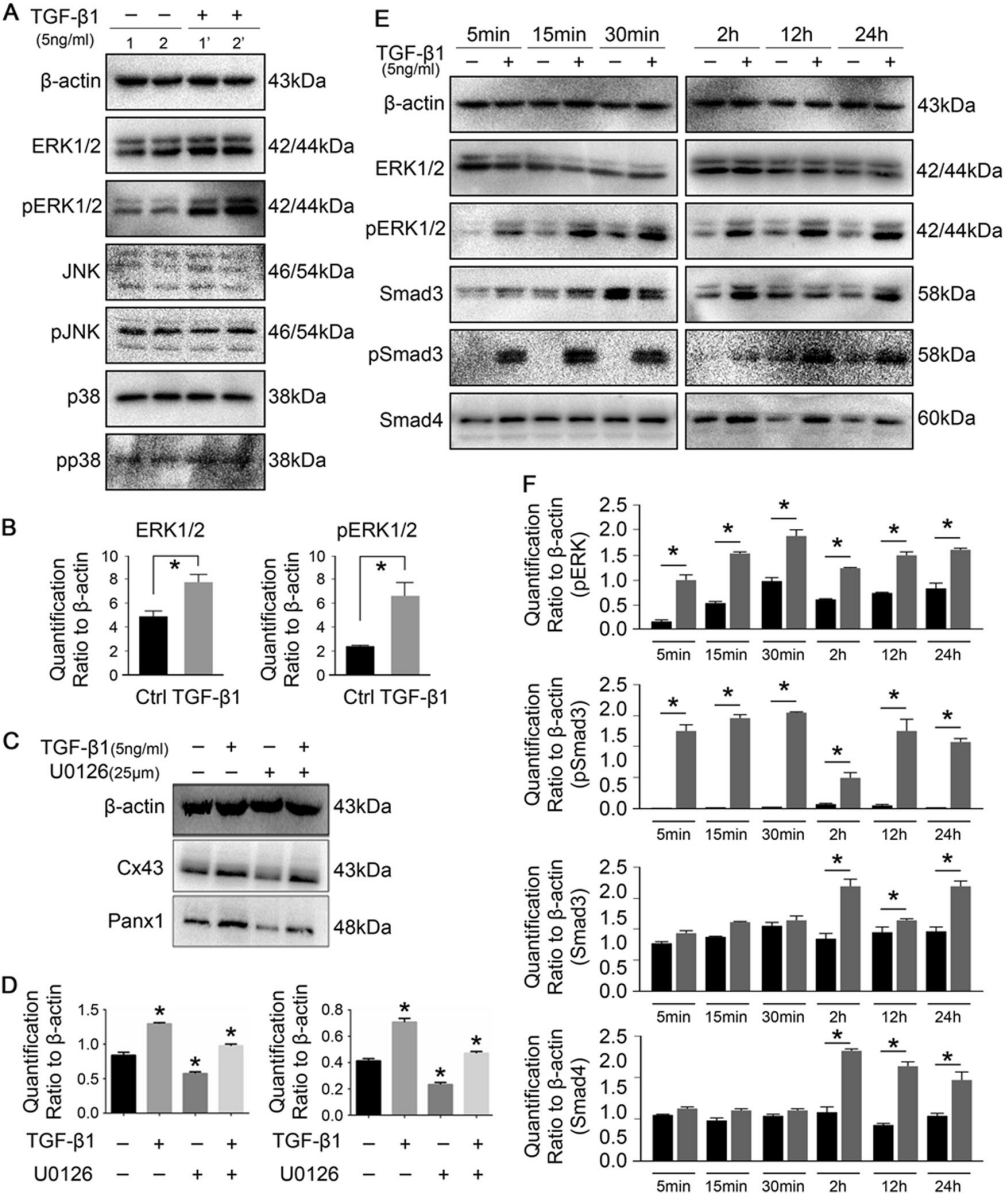
TGF-β1 mediates gap junctions through ERK and Smad3/4 signaling pathways. **a** Representative Western blots showing the different expressions of MAPKs in osteocytes (MLO-Y4 cell line) induced by TGF-β1. Sample 1, 1′ and 2, 2′ are from two independent experiments. **b** Quantitative analysis showing TGF-β1 induced highly expressions of total and phosphorylated ERK1/2 in osteocytes. Data are presented as the mean ± s.d.; *n* = 3, *p < 0.05 by *t*-test. **c** Representative Western blot images of Cx43 and panx1 showing interaction between TGF-β1 and ERK1/2 signaling. The presence of U0126, ERK1/2 inhibitor, reduced the expression of Cx43 and panx1 at 25 μM. While TGF-β1 promoted the expression of Cx43 and panx1 in the presence of U0126. The results shown are based on three independent experiments (*n* = 3). **d** Quantification in **c** was performed to confirm the protein changes. The results were normalized to β-actin as Western blot loading control. Data are presented as the mean ± s.d.; n = 3, *p < 0.05 by *t-*test. **e** Representative Western blots showing protein expressions of total and phosphorylated Smad3 and Smad4 beyond ERK1/2 in osteocytes after treatment with TGF-β1 (5 ng/ml) at different time points. The results were based on three independent experiments (*n* = 3). **f** Quantitative analysis of proteins in **e**. The results were normalized to β-actin as Western blot loading control. Data are presented as the mean ± s.d.; *n* = 3, *p < 0.05 by *t-*test

Previous studies have addressed cross-talk between MAPKs and Smad signaling and the activation of MAPK cascades by TGF-β1, including ERK1/2, JNK1/2 and p38^21^. Here, we aimed to reveal the precise mechanism in TGF-β1-mediated gap junction formation. After treatment with TGF-β1 (5 ng/ml) for 15 min, the activation of ERK (p44/p42) was detected, whereas the expressions of total and phosphorylated JNK and p38 remained unchanged (Fig. 4a). Quantification showed that the total and phosphorylated ERK levels in osteocytes induced by TGF-β1 were enhanced up to 1.7- and 2.8-folds relative to normal control groups, respectively (Fig. 4b). To further confirm the influence of ERK1/2 signaling on Cx43 and panx1, an inhibitor of ERK signaling, U0126, was applied (Fig. 4c). It was found that inhibition of ERK effectively reduced the expression of Cx43 and Panx1. However, in the presence of TGF-β1, the expression of Cx43 and Panx1 was restored and even shown to be higher when compared with normal controls. This result inferred that other signals may have been involved. We detected Smads signaling and found that the expressions of total and phosphorylated Smad3 significantly increased during an initial stage after TGF-β1 treatment (within 30 min) and remained dominant at 24 h (Fig. 4e, Smad3 part). The expression of total Smad4 also increased but was far less prominent compared with that of Smad3 (Fig. 4e, Smad4 part). Although TGF-β1 restored the expressions of Cx43 and Panx1 in the presence of U0126, the activation of phosphorylated ERK1/2 is a continuous process and cannot be ignored (Fig. 4e, f, pERK part). Quantification showed that activation of phosphorylated Smad3 was predominant (Fig. 4f).

**Table 1:** Primer pairs for RT-PCR in this study

mRNA	Primer pairs
GAPDH	Forward: AGGTTGTCTCCTGCGACTTCA
(NM_001289726.1)	Reverse: CCAGGAAATGAGCTTGACAAA
Pannexin1	Forward: CCACGTCCCTACAGACCAAG
(NM_001002005.2)	Reverse: TCGCTGCTCAGGTCCAAATC
Pannexin2	Forward: CTCCCAGGCCACCAAAAACT
(NM_001002005.2)	Reverse: AGTTCGATCCTACCCAAGCC
Pannexin3	Forward: GTCATGCTTCCCTTCCCACA
(NM_172454.2)	Reverse: AGATGGGCAGGCCAAATCAA
Connexin43	Forward: TGCACCTGGGGTGTTCATTT
(NM_010288.3)	Reverse: GCCGCCTAGCTATCCCAAAA
Connexin45	Forward: TCATCCTGGTTGCAACTCCC
(NM_001159382.1)	Reverse: CTCGCCATGCTCCATTTTGG
Connexin26	Forward: CATTTCGGACCAACCCAGGA
(NM_008125.3)	Reverse: TGCCCCAATCCATCTTGTCC
Connexin30	Forward: TACCACTAAGACCTGGCCCT
(NM_001010937.2)	Reverse: CCTGTGGCTTCTTTTAACCGC
Connexin31	Forward: ACCAATGCCCCCATTCCAC
(NM_001160012.1)	Reverse: GCCTCAGGGATGCAAGTTCTA

**Fig. 5:**
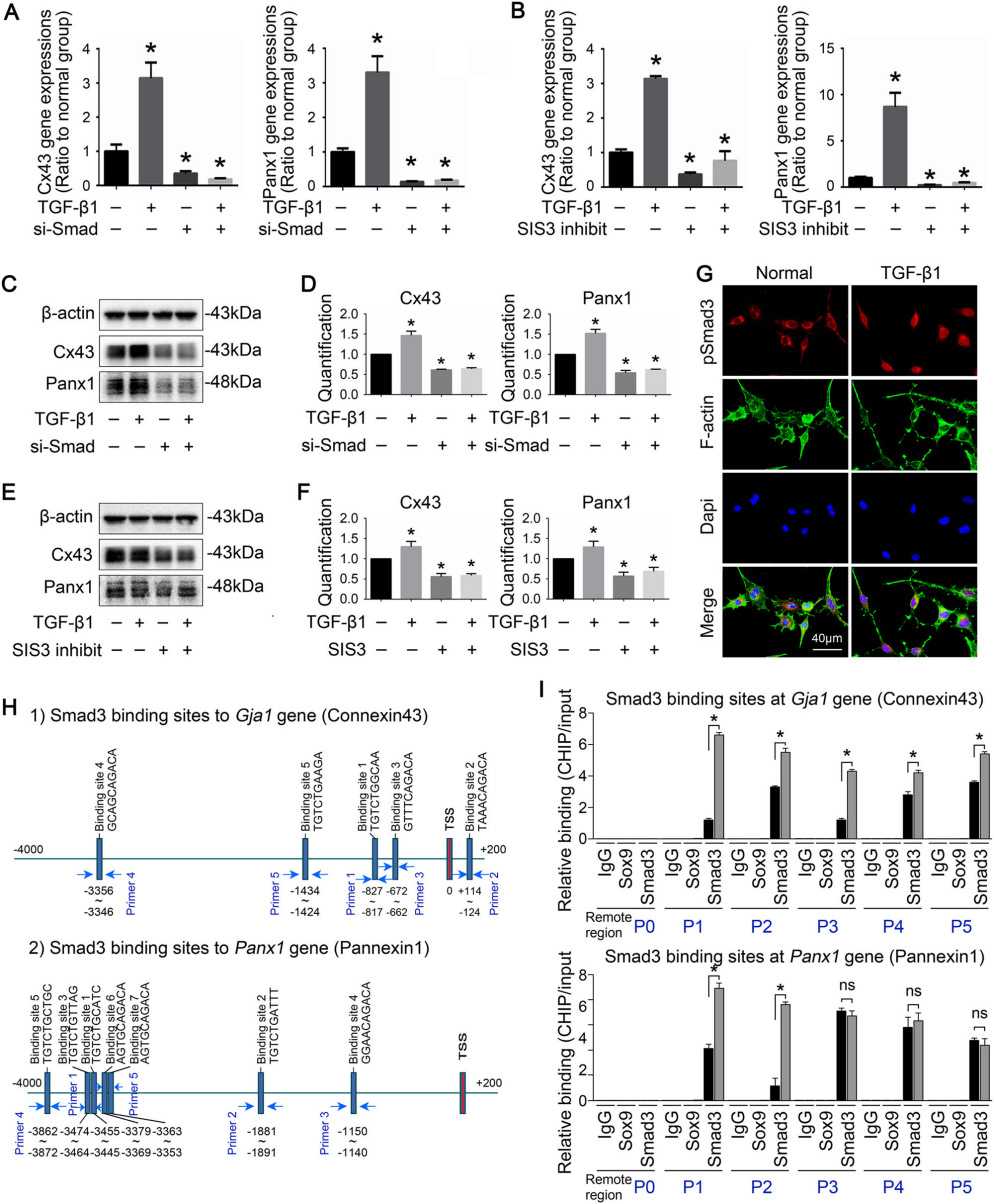
TGF-β1 promotes the translocation of Smad3 into nucleus and the abundant bindings of Smad3 on the promoters of *Gja1* and *Panx1* strengthens gene expressions of Cx43 and panx1. **a** mRNA expression of Cx43 and panx1 in the presence (+) and absence (−) of Smad3 siRNA (100 nM) incubation and subsequent TGF-β1 (5 ng/ml) treatment by qPCR. The results shown are based on three independent experiments (*n* = 3). *p < 0.05. **b** mRNA expression of Cx43 and panx1 in the presence (+) and absence (−) of SIS3 (5 μM) incubation and subsequent TGF-β1 (5 ng/ml) treatment by qPCR. The results shown are based on three independent experiments (*n* = 3). *p < 0.05. **c** Representative Western blots showing protein expressions of Cx43 and panx1 in the presence (+) and absence (−) of Smad3 siRNA incubation and subsequent TGF-β1 treatment. The results shown are based on three independent experiments (*n* = 3). **d** Quantitative analysis of proteins in **c** The results were normalized to β‐actin as western blot loading control. Data are presented as the mean ± s.d.; *n* = 3, *p < 0.05 by *t*-test. **e** Representative Western blots showing protein expressions of Cx43 and panx1 in the presence (+) and absence (−) of SIS3 incubation and subsequent TGF-β1 treatment. The results shown are based on three independent experiments (*n* = 3). **f** Quantitative analysis of proteins in **e**. The results were normalized to β-actin as western blot loading control. Data are presented as the mean ± s.d.; *n* = 3, *p < 0.05 by *t*-test. **g** Representative IF staining by CLSM showing enhanced translocation of Smad3 into nuclei in osteocytes (MLO-Y4 cell line) after treatment of TGF-β1 (5 ng/ml) for 6 h. The results shown are based on three independent experiments (*n* = 3). **h** Bioinformatics showing the predicted binding sites of nuclear translocated-Smad3 on the promoter of Cx43 gene (*Gja1*, GenBank name) and *Panx1* gene. **i** ChIP assay was performed in osteocytes (MLO-Y4 cell line) in the presence or absence of TGF-β1 to confirm the binding sites of Smad3 at the promoter region of *Gja1* and *Panx1* genes. Histogram indicated the relative levels of PCR products at the five binding sites on the proximal promoter of *Gja1* and *panx1* induced by TGF-β1. The assay was repeated at three times (*n* = 3). All data are shown as the mean ± s.d.; *p < 0.05 by *t*-test

To further detect the role of nuclear Smad3 on transcription of *Gja1* and *panx1* gene, we used Smad3 siRNA and SIS3 to reduce Smad3 expression and inhibit Smad3 phosphorylation respectively (Table 2). RT-PCR showed gene expression of Cx43 and panx1 were decreased by reducing expression and phosphorylation of Smad3 (Fig. 5a, b). It was also found that both Smad3 siRNA and SIS3 decreased protein expressions of Cx43 and panx1, abrogated the promoting effect of TGF-β1 on Cx43 and panx1 (Fig. 5c–f). To identify the distribution of Smad3 in osteocytes induced by TGF-β1, we performed immunofluorescence and found that, after TGF-β1 treatment, Smad3 accumulation was detected at the nuclear region (Fig. 5g). These indicated that the activated Smad3 signal may act on nuclei as a transcriptional factor to mediate the expressions of Cx43 and panx1.

**Table 2:** siRNAs sequences in this study

Gene	Strand	Sequence
si-SMAD3	Sense	5′-CAGAACGUGAACACCAAGU-3′
	Antisense	5′-ACUUGGUGUUCACGUUCUG -3′
si-NC	Sense	5′-UUCUCCGAACGUGUCACGU-3′
	Antisense	5′-ACGUGACACGUUCGGAGAA-3′

Using bioinformatics, we predicted potential binding sites of Smad3, i.e., the promoters of *Gja1* (Cx43) and *Panx1* (Fig. 5h). Five and six binding sites of Smad3 were predicted at the promoter regions of *Gja1* (Fig. 5h-(1)) and *Panx1* (Fig. 5h-(2)) (−4000 bp to 0 bp before the transcriptional starting site), respectively. We designed specific primers (supplementary material) and performed ChIP assay to determine the actual binding sites (Fig. 5i). Real-time quantitative PCR (qPCR) based on immunopurified DNA fragments demonstrated that TGF-β1-induced Smad3 greater than controls at all five predicted binding sites on *Gja1*, namely, −3356 to −3346 bp, −1434 to −1424 bp, −827 to −817 bp, and −672 to−662 bp, before the transcriptional starting site (TSS), and +114 to +124 bp after TSS, TGF-β1-induced Smad3 was only enriched at two binding sites, i.e., −1881 to −1891 bp and −3474 to−3455 bp on the *Panx1* promoter. These results indicated the direct modulation of TGF-β1 on *Gja1* and *Panx1* genes. We also showed PCR results based on agarose gel electrophoresis (Fig. S1, in Supplementary material), it also provides an additional support for the ChIP assay results.

## Discussion

Cell differentiation, growth and development depend on cellular responses to extracellular stimuli through communicative channels, including connexin hemichannels and pannexin channels. Connexins and pannexins are two important families of channel-forming proteins. Pannexins have only been recently identified and are far less understood when compared with connexins. It is generally accepted that Panx1 functions as an unpaired, large pore single membrane channel in vivo^22^. Because pannexins are frequently visualized at cell membranes where there are no opposing cells, researchers have suggested the use of term “channels” and not “hemichannels” when referencing to pannexins^4^. In this study, we observed that panx1 mainly localized in the cytoplasm and processes of osteocytes, different from Cx43, which cluster at the tips of processes and form GJs.

In mature bone, the osteocyte body and its processes (cytoplasmic arms extending from osteocyte) reside in spaces and channels called lacunae and canaliculi, respectively. Gap junctions, which are often formed at the tips of osteocyte cell processes, respond to changes in the mechanical environment. These changes are often induced by stimuli, such as mechanical loading, which are transmitted through the osteocyte network^23^. In our previous study, we found that TGF-β1 was relatively highly expressed in osteocytes and bones^24^. We applied recombinant TGF-β1 as an exogenous factor to detemine its role in regulating Cx43 hemichannel and panx1 channel formation. Surprisingly, by SEM we found that TGF-β1 promoted dendritic processes formation, and positively regulated the expression of Cx43 and panx1. We hypothesized that this may be one of the mechanisms by which TGF-β1 promote GJs. However, inhibition of TGF-β signaling by Repsox decreased the number of dendritic processes and increased the number of empty lacunae. Because lacunae provide volume for oxygen and nutrient-rich fluids to maintain the viability of osteocytes, we predicted that Repsox may also affect the homeostasis of osteocytes.

TGF-β receptors include a type II receptor (TβRII) and two type I receptors. One type I receptor is the ubiquitously expressed TβRI, also known as ALK-5, and the other is the more selectively expressed ALK-1^25^. TGF-β first binds to TβRII to form a complex. This complex recruits TβRI by recognizing a unique interface generated by the TGF-β-TβRII complex and triggers downstream signaling cascade^26^. Present in the cytoplasm, R-smad (Smad2 or Smad3) are subsequently phosphorylated by the complex and attract C-smad (Smad4) to form further complexes. Those complexes subsequently translocate into the nuclei to regulate gene expression^27^. To demonstrate the role of TGF-β1 in regulating Cx43 and pannexin1 expression, Repsox, a specific chemical reprogramming tool and ATP-competitive inhibitor of TGF-β receptor 1 kinase (ALK5)^28^, was used to block TGF-β signaling. We used immunofluorescence staining assays and observed Smad3 translocation from the cytoplasm to the nuclei in osteocytes upon TGF-β1 administration. We confirmed the binding and effects of Smad3 to *Gjal* and *panx1* gene. Treatment with TGF-β1 triggered the expression of Cx43 and panx1 and subsequently promoted GJs formation. Therefore, the TGF-β-induced upregulation of Cx43 and panx1 were mediated by Smad3-dependent pathway.

To date, in addition to the canonical Smad-dependent pathway, TGF-β also participates in a non-Smad pathway that includes various branches of mitogen-activated protein kinase (MAPK) pathways^29^. We first revealed that TGF-β1 strongly activated the ERK1/2 signaling pathway in osteoid cells. Activated TβRI has been reported to contain intrinsic tyrosine kinase activity (not only Ser-Thr kinase activity); further, Shc can be directly phosphorylated on tyrosine and serine residues^30^. Phosphorylated Shc combines with TβRI, followed by Grb2 and SOS recruitment, resulting in the activation of Ras-Erk MAPK signaling^31^. As shown in previous studies, the activation of ERK1/2 leads to increased TGF-β1-induced Cx43 expression. We applied loss-of-function approaches using U0126, an ERK kinase inhibitor, to further confirm the roles of ERK1/2 in TGF-β1-induced Cx43 and panx1 increases. As expected, the ERK inhibitor attenuated the upregulation of Cx43 and panx1 by TGF-β1, which confirmed previous reports that the activation of ERK1/2 leads to increased TGF-β1-induced Cx43 expression^17^. Indeed, ERK activation has been shown to enhance Cx43 expression in several cell types^32,33^, and the collaboration between the Erk MAPK and Smad pathways has been reported in many physiological and pathological processes^34^. Thus, ERK could phosphorylate the linker regions of nuclear-localized Smads, enhance Smad-mediated transcriptional activity^35^ and increase the duration of Smad target gene transcription^21^.

In summary, our findings demonstrated that TGF-β1 upregulated Cx43, pannexin1 and GJIC activity in osteocytes. These results corroborate the general agreement that TGF-β1 is a pleiotropic factor and its effect on Cx43 expression depends on cell type. Moreover, the mechanism behind the upregulation of Cx43 and panx1 by TGF-β1 is the activation of Smad3/4 and ERK1/2 and resultant downstream cascade signaling. Our data provides important insight into the molecular mechanisms for cell–cell communication between osteocytes. However, the roles of TGF-β1 on Cx43 and panx1 hemichannel formation require further investigation.

## Materials and methods

### Cell culture

Murine osteocyte-like MLO-Y4 cells (American Type Culture Collection, Mannasas, VA), which has characteristics similarly to primary osteocytes, was used in this study as previously reported^24^. We maintained cells (passage 3–5) in DMEM (high-glucose DMEM, 0.1 mM nonessential amino acids, 4 mM L-glutamine) supplemented with 10% fetal bovine serum (FBS) and 1% penicillin-streptomycin solution. Cells were cultured in six-well plates at a density of 2 × 10^5^ cells per well and were grown at 37 °C in a 5% CO_2_ incubator, starved with DMEM contains 2 and 1% FBS for 12 h respectively and then treated with recombinant mouse TGF-β1 (R&D Systems) and Repsox (ab142139, Abcam, Cambridge, UK) for 48 h.

### RNA extraction and quantitative real-time PCR (RT-qPCR)

Total RNA was extracted from MLO-Y4 cells using the RNeasy Plus mini Kit (Qiagen, CA, USA) according to the manufacturer's recommendations. The RNA samples were reverse-transcribed into cDNA by using the cDNA synthesis kit (K1621-RevertAid, Mbi, MD, USA) according to the recommended instructions. RT-qPCR was carried out with the SYBR Premix Ex Taq II PCR Kit (TAKARA, Shiga, Japan) using an iCycler (Bio-Rad) and each reaction contains 1 μl template cDNA, 1 μM for each primer pairs (Table S1 for pannexins) in a 25 μl volume. The cycle threshold (Ct) values for the samples were normalized to that of *gapdh* and the relative expression was calculated using a ∆∆Ct method.

### Western blotting

Cells were lysed in RIPA buffer (Pierce, Rockford, IL) on ice. The samples were denatured at 100 °C for 5 min with one part of Bio-Rad Laemmli sample buffer (Bio-Rad Laboratories, Hercules, CA). Proteins was separated using a 10% Bis-Tris gel and then transferred to a polyvinylidene fluoride (PVDF). The membranes were blocked for 1 h in Tris-buffered solution containing 0.05% Tween 20 and 5% non-fat dried milk and incubated overnight at 4 °C with the relevant primary antibodies (β-actin, 1:2000, sc-47778, Cx43, 1:3000, #11370; pannexin1, 1:1000, #139715; Smad3, 1:3000, #28379; Smad4, 1: 5000, #40759; p-Smad3, 1:2000, #52903; ERK1/2, 1:1000, #54230; pERK1/2, 1:1000, #201015, Abcam, Cambridge, UK; JNK, 1:1000, #9252; pJNK,1:1000, #4668; p38, 1:1000, #9212S; pp38, 1:1000, #4511S; Cell Signaling Technology), then added corresponding secondary antibody (m-IgG_К_BP-HRP, 1:4000, sc-516102; mouse anti-rabbit IgG-HRP, 1:2000, sc-2357) and incubated for 2 h. β-actin was used as the internal control. The immunocomplexes were visualized by chemiluminescence using an ECL detection kit. Quantitative analysis of the blots was performed using ImageJ software (NIH, Bethesda, MD, USA). All experiments were repeated three times and the most representative images were selected to present in the results section.

### Immunofluorescence staining

Immunofluorescence staining were performed as previously described^36^. Briefly, Cells were cultured in Petri dishes specified for confocal laser microscopy for 12 h. Then TGF-β1 (5 ng/ml, p04202, R&D Systems, USA) and Repsox (50 μM, ab142139, Abcam, Cambridge, UK) were added into the culture media respectively and continued to incubate for 24 h. Then the cells were fixed in 4% paraformaldehyde for 20 min, and rinsed with PBS three times, permeabilized with 0.5% Triton X-100 (Beyotime, Shanghai, China) for 15 min, and blocked with 5% BSA for 1 h. The samples were then incubated with the Anti-Cx43 (1:200; Abcam, Cambridge, UK), pannexin1 (1:200; Abcam, Cambridge, UK), Smad3 (1:200; Abcam, Cambridge, UK) rabbit monoclonal antibodies overnight at 4 °C. The secondary antibody was Alexa Fluor® 647 (10 μg/ml, Alexa Fluor ®647, Life Technology, Grand Island, NY, USA). Nuclei were counterstained with 4′,6-diamidino-2-phenylindole (DAPI; D9542, Sigma, USA) and phalloidine (6 μM, Invitrogen, CA) was used to label the cytoskeleton.

### Acid etching and scanning electron microscopy (SEM)

To evaluate the canalicular network in the cortical bone and quantify the number of dendritic processes elongating from osteocytes, acid etching was performed on mouse femur, as previously described^37^. Briefly, the bones were dissected from the tendon and muscle attachments, preserve both ends of the femur. Bone samples were incubated in DMEM containing 10 ng/ml TGF-β1 and 100 μM Repsox respectively for 21 days with the culture media changed every two days. Then the bone specimens were then fixed in 70% ethanol and non-decalcified samples were embedded in polymethyl metacrylate (PMMA). The PMMA embedded samples were polished using an automatic grinding system (Exakt, Germany) to obtain a coplanar plane. Polished samples were soaked in 9% phosphoric acid solution for 20 s (polished side upwards), rinsed with deionized water (1–2s) and incubated in 5% sodium hypochlorite for 5 min, and dried at room temperature. Specimens were then sputter coated with a gold alloy and scanned by scanning electron microscopy.

### Scanning electron microscope (SEM) test

For observation of morphological changes of MLO-Y4 cells, the cells were seeded onto Petri dish, cultured and serum starved, followed with treatment with TGF-β1 (5 ng/ml) and Repsox (50 μM) for 48 h. The culture medium was discarded and PBS was used to wash the samples three times. Cells were then fixed with 2.5% glutaraldehyde for 2 h and then dehydrated in graded ethanol with concentration from 50%, 60%, 70%, 80%, 90%, 95%, to 100%. After dehydration, the samples were placed on specimen holders, coated with a thin layer of gold, and then imaged by scanning electron microscope (SEM).

### Scrape loading and dye transfer assay

To examine the effect of TGF-β1 on the intercellular communication between osteocytes, we applied the scrape loading and dye transfer (SL/DT) assay, which has been recognized as the most commonly used assay to measure intercellular communication^38^. This technique relies on introducing small molecular (MW < 900) dyes (Lucifer Yellow, MW457, L0259, Sigma) and tracking their intercellular movement through gap junctions. Briefly, the adherent cells were treated with TGF-β1 for 6 h as experimental group. After treatment, fully confluent cells were washed with CaMg-PBS and scraped using a surgical blade prior to the addition of fluorescent dye (1 mg/ml Lucifer Yellow). After incubation for 2 min at room temperature, the cells were rinsed thoroughly to eliminate background, and images were immediately collected as baseline. We monitored the transfer of the LY dye through several adjacent cell layers for 7 min.

### HE staining and IHC

Histopathological examinations were performed according to routine steps^39^. The femurs were collected as above, then decalcified with 15% EDTA for two weeks and hydrated with graded ethanol (100–70%), 5 μm longitudinal sections were stained with hematoxylin solution for 5 min followed by 5 dips in 1% acid ethanol (1% HCl in 70% ethanol) and then rinsed with distilled water. Eosin solution was used to stain the sections with for 3 min followed by dehydration with graded alcohol and clearing in xylene.

As for Immunohistochemical staining, bone sections were harvested as above. To prepare slides of osteocyte, cells were directly seeded on glass coverslips and treated with TGF-β1 (5 ng/ml) for 24 h. For tissue sections, the slides were treated in sodium citrate buffer (10 mM Sodium citrate, 0.05% Tween 20, pH 6.0) at 90 °C for 15 min for antigen retrieval and endogenous peroxidase activity was inhibited by incubation with 3% H_2_O_2_ for 30 min, the slides and coverslips were rinsed and incubated with the primary antibody (Cx43, 1:200) overnight at 4 °C. The next day, the samples were rinsed and incubated with the second antibody (Beijing Biosynthesis Biotechnology Co. Ltd.; Beijing, China) for 2 h followed by DAB and hematoxylin staining. The cell slides were fixed with acetone for 10 min at 4 °C, dried at RT and rinsed with PBST (PBS with 0.1% Tween 20, pH 7.6) prior to the following procedure as above. The slides were then photographed using an Olympus BX53 fluorescence microscope (Tokyo, Japan).

### Small interfering RNA (siRNA) transfection and Smad3 phosphorylation inhibition

To knock down Smad3 expression, cells were transfected with 100 nM siRNA that targets specific gene (Dharmacon) using Lipofectamine RNAiMAX (Invitrogen, Burlington, ON, Canada). Scrambled-sequence siRNA was used as a control in all experiments (Dharmacon). To inhibit Smad3 phosphorylation, specific inhibitor of Smad3 (SIS3, B6096, APExBIO, USA), which selectively abrogates Smad3 phosphorylation, was used to preincubate cells in 5 μM for 6 h before TGF-β1 (5 ng/ml) treatment for 24 h.

### Chromatin immunoprecipitation (ChIP)

Chromatin immunoprecipitation was performed using Pierce^TM^ Agarose ChIP Kit (Lot#TA265476). Briefly, the cells were fixed with 1% formaldehyde and enzymatically digested by micrococcal nuclease (MNase) to 200–500 bp on ice. One-tenth of the total cell lysate was used as the DNA input control. The remaining of the total lysate were subjected to immunoprecipitations with anti-Smad3 antibody (ab28379, Abcam, UK), anti-SOX9 antibody (ab3697, Abcam) and non-immune rabbit IgG. Protein–DNA complexes are stabilized and then extracted according to the manufacturer’s instruction in the Kit. Crosslinking performed directly in cells locks in the protein–DNA complexes, trapping these unstable and sometimes transient interactions. The DNA fragments which was pull out by protein–DNA complexes after overnight incubation with Smad3 antibody was used as the template for qPCR. The primers targeted for binding sites of Cx43 and pannexin1 were designed and the specificity was checked by BLAST tool in NCBI. Information about detailed promoter binding sites and primer design are in Supplementary material. PCR products were analyzed by 2% agarose gel electrophoresis.

### Statistical analysis

The results are presented as the mean ± s.d. of at least three individual experiments and plotted with GraphPad Prism (Inc., San Diego, CA, USA). One-way analysis of variance (ANOVA) was used for multiple comparisons, and Student’s *t*-test was used to determine significant differences between two sets of data. The critical significance level was set to be *p* < 0.05.

## Supplementary information

Supplementary materials

## Data Availability

The authors declare that the data supporting the findings of this study are available within the article.
