# Supplementary material for: TGF-β1 facilitates cell–cell communication in osteocytes via connexin43- and pannexin1-dependent gap junctions
Source: Cell Death Discov. 2019 Oct 25;5:141. doi: 10.1038/s41420-019-0221-3 (PMC6814792; doi:10.1038/s41420-019-0221-3)
Supplement: Supplementary file 1 — Supplementary materials [file 41420_2019_221_MOESM1_ESM.doc]

**Supplementary materials**

1. Supplementary figure

Figure.S1

**
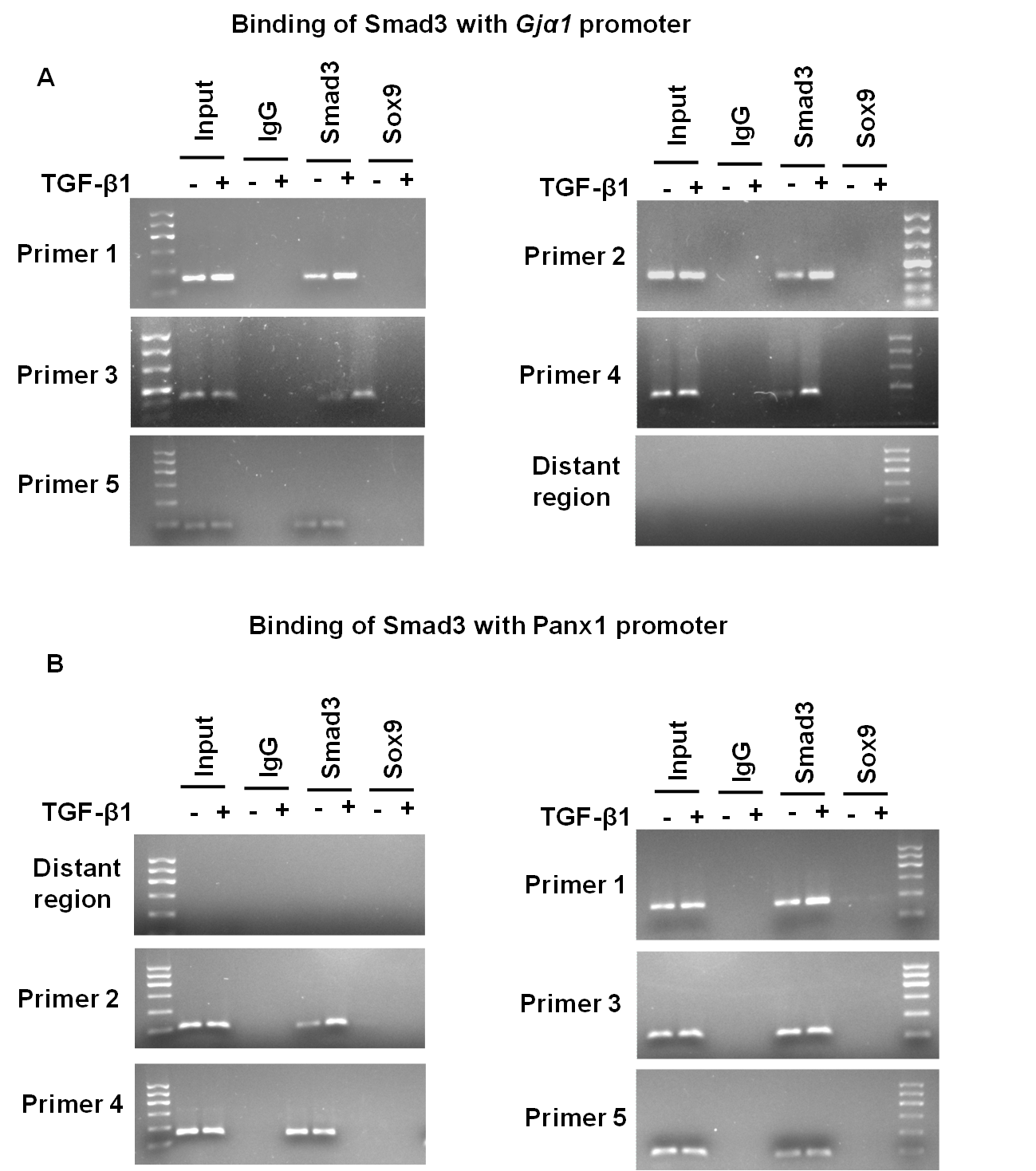
**

**Figure.S1** PCR results by agarose electrophoresis showing the Bindings of Smad3 in the promoter regions of *Gjα1* and *panx1* by ChIP. (A). TGF-β1 induces Smad3 enrichment over the control at predicted binding sites on *Gjα1.* (B). TGF-β1 induces Smad3 enrichment over the control at predicted binding sites on *Panx1.* Left panel: Lane 1, DNA marker; lane 2-3, PCR product derived from direct input DNA template in the absence (−) and presence (+) of TGF-β1; lane 4-5, PCR product derived from DNA template immunoprecipitated by IgG in the absence (−) and presence (+) of TGF-β1; lane 6-7, PCR product derived from DNA template immunoprecipitated by Smad3 in the absence (−) and presence (+) of TGF-β1; lane 8-9, PCR product derived from DNA template immunoprecipitated by SOX9 in the absence (−) and presence (+) of TGF-β1. Right panel: Lane1-2, PCR product derived from direct input DNA template in the absence (−) and presence (+) of TGF-β1; lane3-4, PCR product derived from DNA template immunoprecipitated by IgG in the absence (−) and presence (+) of TGF-β1; lane5-6, PCR product derived from DNA template immunoprecipitated by Smad3 in the absence (−) and presence (+) of TGF-β1; lane7-8, PCR product derived from DNA template immunoprecipitated by SOX9 in the absence (−) and presence (+) of TGF-β1; Lane 9, DNA marker.

**2. Supplementary material**

**-Supplementary information about detailed promoter binding sites and primer design**

1. The detailed information about ***Smad3*** at the promoter of **Connexin 43** (gap junction protein alpha 1, ***Gjα1*** in mice):

The sequences in capitals at the promoter of ***Gjα1*** (Gene ID: 14609, Chromosome 10 - NC_000076.6, **-4000** bp in capitals ~ **+200** bp in grey (Transcriptional starting site, TSS))

**Smad3: TOP five binding sites at *Gjα1***

1. TGTCTGGCAA Dissimilarity: 1.82% -827 ~ -817

2. TAAACAGACA Dissimilarity: 2.20% +114 ~ +124

3. GTTTCAGACA Dissimilarity: 4.08% -672 ~ -662

4. GCAGCAGACA Dissimilarity:4.17% -3356 ~ -3346

5. TGTCTGAAGA Dissimilarity: 4.33% -1434 ~ -1424

**Primers designed for detecting these binding sites:**

1. Primer pair 1 (88 bp): Forward: GAAAGTGCTTCCGAGGGACA

Reverse: TTCTAACCGCTGCAGACCAG

2. Primer pair 2 (96 bp): Forward: AGTACCCAACAGCAGCAGAC

Reverse: GTGGAACTCCTTGGAGGCTG

3. Primer pair 3 (170 bp): Forward: CCTTGGAGATTGGGTTGAGGT

Reverse: CCGTGGAGTGGGATGTCTAC

4. Primer pair 4 (158 bp): Forward: CCAGGATAGCTGCTCACACC

Reverse: GCTTGTCTGCTGCCAGTTTT

5. Primer pair 5 (110 bp): Forward: CTCTTCTGGTGTGTCTGAAGAGT

Reverse: CTTTATGTCCAGCAATGAATGTGAT

6. Primer pair (74 bp): Forward: AACTCCAGCTCCTTGCCTTC

Distant region Reverse: GCCTGTATTGCCTCACGACT

The primer sequences are corresponding to those of sequences in binding sites.

**The sites at the promoter region of** ***Gjα1***

AAAGCAAAACAAAGCAAATTCTTCCTTCCCTTTATAAACATGTTGTTTGTTTGTTTGTTTAGCTTCAAAGTCAGATTCACTAGGAGGTAAATGGAAGACCCATAGAAATTTTACCTAGACAGAGTCAAATTTTGACGTTTACATTGTTTTCAAAGATGTCAGAGAGCTAAATAAATGGGGAGCAAGGTTTCAGTTACAGGAGTCCTCTGGAATTGAATAGGAGGTGAGTTGTCAGGTTTCAGAAACTATGTAGAAAAGGATTCTAGTGTAATTTGTTTGTGAAGCTGCTGGCTCCTAGCATTCACAGTAAGATCCCTTCCCAGCTGGCTACCAATCTAGGAAAAGCATTCCCATATTTGCAAGCCGTCAAGCTCCACATTCTCGTCCACTCTTTCCTTGCAGACGGAAAAGAACACACAAAAAAAAAAAAAAAAAGTTAGCTTAAATTGAAATTTGATCCATACAAAATTATTGTAGTTCTGATAGAAGAAAATATAAACCAGGATAGCTGCTCACACCAGCATTCTAACACAGGAAATAAGGGAGGAGGGTCATAACTTGTTTAGTGAGACTTTATCTCAAAATTTAAAACAAACAAAAAAAAAACCTCAACCTCAGATAAAGGTGTCTTTAATCAAAAACTGGCAGCAGACAAGCTGGGGATCTATATTGAGACTTTAACTCAAGAAGGAGCTGGAGGAGAAGGAGGAGGAAGAGGAGGAGGAGAGAGGAGAGGAAGAAAGGAAAGGAAAGGATAAGATAATAAAATCTAAGCCCACTGCAATGTTATTCTTGATCCTCCCAGTCTTAGATAGCTCTGTCTCCTACAGATCCATCTTCATTCCCAAGGAGCTACTTATCAGCTCTTCAGTCAGGTGACCACCTCTCTCTTGGGAGAGAAACTACCTTCATTCTCCTACATCCCAGCCCTCTACCTCAGCACTTCATTTTCTGTAAGGTCTGCATTCACTCTTGTTTTCAATGAATAATTGCCCTTTCTGAGTTAAAGACCTAGAAAGCCTCAGGCTAGTGTTATTTTCCTTACAACTAAACCGCCTCCCAACCATCTTGAATGCCTTTCTCCTCTTGACCTTACTGGCTTTTGTCATTCTTTTCTTTGTGGGCTTTAGAGGAAATCCATTCAAGCTGTGCGCTTTGTCTTGGAGATTCACGGCCTACATTCCTTCTTTTAACAAACACTTTAGAACCTGTGGCTTTAACTGTCGATTCTTTTCCGTTAAATTACAATCTTTACAAAATTTTAACCTGAACACCTTAACCCACAGTTGAGAGGCCCCTTGGCTACCTTTGCCTAGGCATTTACGCTCTCCATCACTGGATTCTCTCTCCTATAGTGTCTTCTGCTGCCTCCACCCCTTCCCTTACCCCCCACCCCGCACCCCTGCACCCCAGCTTCCGTTTTTGGTGTTAGTAATATTTTCCCAATTAAATTTCCAAACTTGCTCTCCACTTCCTCCTGCCTCTTGTTTTCCTGACCTATCAGTCCATGAACAAGCTCTAACGTCTCTGCTTCCCTTTCTTCTCTAAGGTTGGCACATTCATAGCTCACCTCTCTTCCATTCTAGCACCAACTGCGATGCTCTCCATAACAGTCTTTACAGCCTGTTTTTGTTTTTTAACTCAACACTGGGTTGTCTCTAATGCAGTTGAGGATTACTTGAAACTCTTCTCAGATGATTGTACTGTGTTACCTTCTCAGGTGACTGGGTCTATAGGCAAAGACCATTGGGATTAAAGGTGTGTGCCCCATGCCCAGCAAATCTTTCTTTAAATTAAATAAAATTTGGTTTAGTAAAGAGGGAAAGAGGAACAGGTAGACTCGTGAACACTAAGTTCTACTGAGGTTCTATAAATGTGCGTAAGCTAACTATGGGTGGATGATGAATTGTTTGCTTCTAAAAACAATTCTTTAGAAGTCTGGATATTTTCCCATAGAGAATAATAAATATTACAACTGACAAATTGCTTATGCTGAGTTCTATACCACAAACTTTACACTTGTATCAAAACATCACATAACCCCCAACATTACATACAATTCTTATGTGTTTTATCAACTAAAAATAAATTTAATATGCCAGGCATGTGGTGCGTACATTTAATCCCAGTGCTGAAGAGGCAAAGGCAGGCAGAACTCTGTGAGTTCGAGTTCCAGGACGGCTACAACTATGTAGAGACCTCGTCCTTTAAAAAAAAAAAAAAAAAAAAAAAAAGTAACTAAATAAATTTAATAAGAACCAACAAGCTAGCTCAGTGGTAAAGGTGCTGTTTTCAGGCTGAGTATCTAAGTTAATTTTGAGATGGAAAAAGGGATCCAGTTTCACAAGCTGTCTTCTAATTTCCACTTGCATACACATATATGAATACACATAATAAATAAAGGTTAAAAAAAAACCAAAAACAAAAACAGAAACTCTAGGAGCTGGAGAGATGGCTCAGTGATTAAGAGCACTAACTGCTCTTCCAGAAGTCCTGAGTTCAATTCCCAGCAACCACATGTTGGCTCACAGCCATCTGTAATGAGATCTAAAGCCCTCTTCTGGTGTGTCTGAAGAGTTACAGTGTATTTATATACATAAAATAAATAAATCTTTTTTTAAAAAAATCTAATGTCACATTATCACATTCATTGCTGGACATAAAGCCTTGAAAGTCCGATTATAGTTTCTGCATCTGTAACATTACTTCCCTTCTTTAAGTTAAAGACAAAGGAACCTCAAAAGTAAGAGAACATGCTGGGGTCTTAAACTGTGAGGTTTTAAATGGCACTCCCTTAGTCATCCTAGTGAGAGGGTGCTTCGGCAGGAAAATGTTTCCCAGGAAGTCTTCGCCATCCTTTGTTCTGCTATTTACATTAGTGTGAGACGTTCAGCTCCATCAGGTGATGCCTATACAAAGGCCTAACTCCAGGAGCGCCGCTGAAGCCTGTTTATCGCCCTATCACAAGCCCTCGTGGGGGGCATCTGGGAACCCTCAAAGGAGCTGCCCACCCTAGGAATGAGGTCATATGACTCAGGCTTTCATAAACCCCATCAGGTATGATGAGAGAGGCATGAAGCTGTGGGCAGGCAGTCACCAGGCCCACATCTCTTCCCTGAAAGTGCTTCCGAGGGACAGGATTGGAAGAGAGAAAGAAGTTTTGAGACAGGCTGTGTCTGGCAACTCTGGTCTGCAGCGGTTAGAAGGAAGAGGAGGAGGGGGACAATTCTGGCATCTCCCTGCCGATTTAAATGTTAAATTGTAGGCATAAAAAAGACTAAGATATGGATCTTCACTTCCTTGGAGATTGGGTTGAGGTCCTATCAACGTTTCAGACAAGAGTTACATACATGATATATTATTTTTATTCATAGTATAGAACATGTATCTTCTTGTTGTTAATTAAAAGAATAAGGAAAACTACCTAAAGCAGCAGAGAAACTGAAGAGTAGACATCCCACTCCACGGGTCTGGTTGTGAAATGCCTTTCCCATGCCCACCGCCTCTTTCCTTTGTTAATTGTCCGGGTTTAAGAGGAAGAGAATTAGGTCATAAGAAAGATCACTCACACCCACCTCAAGCTTCACACACCTTGGGCAACCCAGAACAAGTCTGCACGAGTTCACCTTGTCTCCCCCCCCATTTTTATTTATTGTAATATTATTATTATTATCATTATTATTTAGGAAATGTGACCTAAAAGGGACATCTTCTCACTGCCCGTGGTCATCTCCTGAAGGAATGACCCATCCAACAGTTTTTTTTTTTAATCTGTGAGGAGTCACAGCCCCGCAGTAGCTGACGTACATCTAGAGCTATTTCTTACTTTTTTTCCCCTTCTCGTCAGCACATTGAAACTACAACTTTATCTTGACCAGGTTGCTTCTTCTGCCTGCTCAGCTCCACGCTCGCCAGCCTCCACTCCACCTCCTCCCCGCCTTTTCTTCCTCCCTCCCCTTTCTCCTAGCCCCTCCTTCCAGTTGAGTCAGTGGCTTGAAACTTTTAAAAGCTCTGTGCTCCAAGTTAAAAAACGCTTTTACGAGGTATCAGCACTTTTCTTTCATTGGGGGAAAGGCGTGAGGGAAGTACCCAACAGCAGCAGACTTTGAAACTTTAAACAGACAGGTCTGAGAGCCCGAACTCTCCTTTTCCTTTGACTTCAGCCTCCAAGGAGTTCCACCACTTTGGCGTGCCGGCTTC

2. The detailed information about **Smad3** at the promoter of **Pannexin1** (*Panx1* in mice):

The sequences in capitals at the promoter of *Panx1* (Gene ID: 55991, Chromosome 9 - NC_000075.6, **-4000** bp in capitals ~ **+200** bp in grey (Transcriptional starting site, TSS))

**Smad3: TOP seven binding sites at *Panx1***

1. TGTCTGCATC Dissimilarity: 2.92% -3455 ~ -3445

2. TGTCTGATTT Dissimilarity: 3.20% -1891 ~ -1881

3. TGTCTGTTAG Dissimilarity: 3.26% -3474 ~ -3464

4. GGAACAGACA Dissimilarity: 3.83% -1150 ~ -1140

5. TGTCTGCTGC Dissimilarity: 4.17% -3872 ~ -3862

6. AGTGCAGACA Dissimilarity: 4.55% -3379 ~ -3369

7. AGTGCAGACA Dissimilarity: 4.55% -3363 ~ -3353

**Primers designed for detecting these binding sites:**

1. Primer pair 1 (117 bp): Forward: CGTCTTCTCCTTTGTAGCACG

Reverse: AGTACGGGTCAGAAGGTCTC

This primer contains No.1 & 3 binding sites above.

2. Primer pair 2 (184 bp): Forward: ACGAACTTAGGCACTGGGC

Reverse: AGTAAAGAAAGCCTCCGACCG

This primer contains No.2 binding sites above.

3. Primer pair 3 (88 bp): Forward: AGTACCCAACAGCAGCAGAC

Reverse: GTGGAACTCCTTGGAGGCTG

This primer contains No.4 binding sites above.

4. Primer pair 4 (179 bp): Forward: TCCCTGCACAAAGCCAATCT

Reverse: CAGGAAGCTGGAGAAGTGGG

This primer contains No.5 binding sites above.

5. Primer pair 5 (106 bp): Forward: GACCTTCTGACCCGTACTGC

Reverse: TCGCTGGCGTACTATTTGCT

This primer contains No.6 & 7 binding sites above.

6. Primer pair (81bp): Forward: TGTTTGCAATATTTGCTTTTCACAG

Distant region Reverse: AGAGAGAGACAGAAACCACGGA

**The sites at the promoter region of *Panx1***

ACTTCACCTGTATGCTAATTCAGAATGTGTTCTCCAACCTTGGCTTTGATCGCAGGTCCACAGGTTTGGCAGGGTCATTGCAGCAGCTCCCTGCACAAAGCCAATCTTGTCCCTTCCATCCACAAAGCTGTCTGCTGCTATGCTCCCCCAGATCTGATCAGGTCACTTCCTGGCGAGTACTATAACCTCTTCCATTATACTCCAGTGCTCCAGACTCTGGGTCTCTGTGGCCTGGTGAAGGCTGTGCCCACTTCTCCAGCTTCCTGGGTTCTCTGCTCCAGGAACTCCTGGGAGTTCTTGCCTCGTTCGGGCCTGTGCACACTCTCCTCTCCATGCCCTTTAATCTGTTCCCTTTGCCCTCTCTGCATTCCACTCCTTTGCCCTCTCCGCATCCCACTTTTCTCCCTTAGGGTTTCTGCTTTAGACAAGAGTTTCTCAGACTGCCTTTTCCAATGTGACCCCCCACTTCTGTTCTGTCCCATCTCCCGTCTTCTCCTTTGTAGCACGAATCAAAGCATGTAATTTCTGTCTGTTAGCTCGAGTTTTGTCTGCATCTTGGCTGGCGTGAAGGTTCCCTGGAAGTGAGACCTTCTGACCCGTACTGCTCGCTGCTGCACCTACAGTGCAGACATCATACAGTGCAGACATCATACAGTGCAGGGCAGTCGCTCAGCAAATAGTACGCCAGCGAGTACTTGAACATTCACACAGCGCAGCCAGTCTGGTGAGCTTGACTATTTTGGCTTTGACTGTTCTCATTGCTAAAGATCTTGAAATCAGATGGCAAGCATGGCAAGCAAGCAAAACCAAGTTTGGGGAGGCTGGCTGTAAGAGCAACCCCCCCCCCCCCATGGATCACTCTGTAGCCAAGAAGGGATCGGGGCAGGGAAGACTTGATGTCCCCAATGCTCCTTATAATTATCAGAAGGCAATGTCATAAAAAAATAAATAAAAACAGAGGACATGAGAAATGTGCCCCCCACCACATGCACAAATATTTCTGTAGTGGTTCATTATTGGAACCAATGCACAATTCTATTGCAAGATGGCACTGAAGAACTTGAATAGAGGAGTGTGGGTATATGAGAACTCTGAATCATTTATTTGCCCACATTTTCTGAAAATCTGAAAACAAACTAAAATCAAATTGAAATCTGGAGTTAGGGAGATGGTTCATTGGCAAAAGGATTGTCATACAAATATGAGGGCCTGTGTGCAGCAATGAAAGGAGCCGGGTATGTCACGTGGCCGTGAGGGCAGCTGGGGAGATAGGGGCAAGGGGGAACCTGCCACTCGCTGGCTAGCTTGTCTTGCTCCCCGAGCTAATGAGCTCCAGCTTCAGAGAGCAAGAGACCTTGTCTCAAAAATGAAATAAAAGAAAAGAAAAGAAAGTAAAATAAAGCCGGGTGTGGTGGCACATGCCTTTAACCCTAGTACTTGGGAGGCAGAGGCAGGCGGATTTCTGAGTTCGAGGCCAGCCTGGTCTACAGAGTAAGTTCCAGGACAGCCAGGGCTACACAGAGAAACCCTGTCTCGAAAAAAAAAGAAAGAAAGAAAGAAAGAAAGAAAGAAAGAAAGAAAGAAAGAGAGAGAAAGTAAAATAAAATAAGCTGGGGAGTGATAGAGGAATATTGCTCACATAGACCTCTGCTTTCCACACAGAGACAAACTTTACACTAAAACCTAGGGTCTATTTTGAAAAATAAAGTAAAACACTTTGGCCATGCTTTCACTTGACGTGTTAGAGTCCTGAGATTATTAATGGGAATGGTGTACAATTCAGGAAATGTTGTTAATAAAATAACACACCCTTATTTGATAGTCTTATGTAGCCCAGGATGCCCAAAATTCAGTGTGTAGCCATAAGTGACCTTGAGCTCTGGGCCTCCTGTCTTTACCTCTCAAGTGCTGGGACTGCCAATGTGTGTCACTACACTCCTCTGGTATTCCGTTGGATTCTAAGAACAATCTGAGGGGACCCTATCGAACCATCCTTATTTGGGTGACAGACCGAGGCTTAAGAAACGAACTTAGGCACTGGGCTCATGGCTTTTACCTTCTGTTTTCCTCCCAGGAATGTTGGCTCAAGCCAGAGATTTTTTTTTTTTTTGTCTGATTTTTTTTGAGTAAGGAAAATGGTTCCCTCAGCTCTCATACCCAGCTCTTGTACCCATTTGTTTCTACCATCGGTCGGAGGCTTTCTTTACTTCTCCTGCACACCTTGGAAGCCTCATTGCTTCAGCTCTCCTTCCACGCCCCTCCACACACTGTTGAAGAGCAAGGGCTTTAAAGCCCAACTCTGTACAGCACTTCCCTCTGCCACTCCCTACAAGTTCAGACTATGCTCCGGCCCCACCTTCCATCATTGACCCCACACCAGTGCCAGGCGTCTCTTCTTCCCAGTTCCCAACAGCTTCTCTTTATTCTCTCTTAAGCTCATCAGTCTTTGACTGATGTTTTAATGAACGAACAAAACTGCCAGGGTGGGGGAAGTTTATAAAACTACTGCGAGACCCAACCGAGAGCCTCAGAAGTCCATGTGTGATCACTGAACAGATACGCAACCCACTATCACCGGCCCGGCCCAGAGTGAGCTGGGTGTTAGAGAACTGGGGGCACCAAGAGAGTGCCTGACTCATCCCAACTAGGGGAGGGGTCATGCGAGGCTTCTCAAAGGGGCCATTTCTCCTCTTGGATGAGTTGATCTTGGCAGAAAGAGAAGAGAAACTGCGTGTGTGACTTACTGGTTCCTAGAAAATACTCAGGACAGTTCTGCCTGCCCCAAACAGACTCGTCCCCTCAGGCTGTGAAACCCAGTGTCACCTCAATCCCATGCCACATGCCTTTCGAGGAACAGACAGGAGTGGGAACTGGGTCACTTAAAAACCACTCAAGGTGAAAGAGATTCACTTAGGACCGTCTGAGTAAAATTAATTAAAGCATGCAAGTACCACTTTTATCTCCCAGCACTCCATAGCCATCTGGAGAAAGATTAGGAGGGTCTGGAGGATGCCTTCCTGTAAGCAGTTAGGGATGGGTGGGTAAATTGGGTGCTGGTTGCTGGCTGTGCAGGTGGGGAACGGTCAAGTCTGCCATATTTAGAAAAGAGAAGCACATCAATTCAGAATTGGTTGGCACAGCTGTCAAGAGTGATGGAAACTCTAAAAATGGAGTACCCGGCTTCTACTTTGGGTGCCAGCACCAACAACAATGGTTTGGGAAAGGTAGAAGACAGTAGTTGAGGTCTGAGAAAGGATTGTGTGAAGCATTGTCAGCACAGGACTGCGAAGGTCTCTGATGGTCTTTAGAGGTCCAGTATGTGGGCCTTGGAGTTACCCTACTCAGACCCCGCTGATGTACACAGCCCAGGGAAGGCGTCTATGCTTGGCACATTCACCTTCCAAGGCCGTTTGCCGTAAATGGTTGGAACATTTCTTGGATGCTTCATTGTCTACTGTAGGTGAGGTATTCAGAAGGAGCTCACCAACACCACATACACAGCAGTTGGCGCCTCAGGAACCATGACTACTGCTTTAGACAGGAAGGTAGAGGAAAGGGCAAAGGGCTGGGCATGCCCTCCCCTTACAGAACTCTGGAAAGATGCAGGCCCTCTCTACAATATACTTTCGGTCATCACCGCAAGATTATCTCGACCTTTTTCTGACACCCGTTGTTTTTGAAGGTTTTTTTTTTTGTTGTTGTTGTTGTTGTTGTTTTGTTTTGAACAGAACCCGGATTGCACCCTGGAGTGTGGCGCTGTGGTTGAAGCGCTTGTCTAGCGTGCAGAGTTGCACAAGACATTGGTTCATCTTCAGCATCAACGCTCTCAATGGTGGACAAGGCTTAGAAAGGGGCTAGCTCGTGCTCCGGGGAGGGAGGCTGTCAGCAGAAAGGAGTGCAAAACCACACACACACGGCTCTAGGGTCTTGTGCAGCCGCGCCCACCTCGCCCGCGTCAGCGCGCTTCCGGAAACTGCTCGCCCCTGGGAACCCGGTTTTCCCCGCGTAGGTTCCGGAAGAGCTCGGTTCTGTAGCCGAGCGCGCAGGGCGGGCGCGGGCAGCTGTAGCGAAGAGCTTTGTTCCCGGCACGTTGTTGCCCCTGCGCGCTCGGAGTGGCCGGAGGGAGTCC
